# Implications of variable late Cenozoic surface uplift across the Peruvian central Andes

**DOI:** 10.1038/s41598-019-41257-3

**Published:** 2019-03-19

**Authors:** Kurt E. Sundell, Joel E. Saylor, Thomas J. Lapen, Brian K. Horton

**Affiliations:** 1 Department of Earth and Atmospheric Sciences, University of Houston, Houston, Texas USA; 2 Present Address: Department of Geosciences, University of Arizona, Tucson, AZ 85721 USA; 3 Department of Geological Sciences and Institute for Geophysics, University of Texas at Austin, Austin, TX 78712 USA

## Abstract

Changes in Earth’s surface elevation can be linked to the geodynamic processes that drive surface uplift, which in turn modulate regional climate patterns. We document hydrogen isotopic compositions of hydrated volcanic glasses and modern stream waters to determine late Cenozoic surface uplift across the Peruvian central Andes. Modern water isotopic compositions reproduce mean catchment elevations to a precision better than ±500 m (1σ). Glass isotopic data show a spatiotemporally variable transition from isotopically heavy to isotopically light compositions. The latter are consistent with modern water on the plateau. When interpreted in the context of published paleoelevation estimates and independent geological information, the isotopic data indicate that elevation rapidly increased by 2–2.5 km from 20–17 Ma in the central Western Cordillera, and from 15–10 Ma in the southern Western Cordillera and Altiplano; these patterns are consistent with foundering of mantle lithosphere via Rayleigh-Taylor instability. The Eastern Cordillera was slowly elevated 1.5–2 km between 25 and 10 Ma, a rate consistent with crustal shortening as the dominant driver of surface uplift. The Ayacucho region attained modern elevation by ~22 Ma. The timing of orographic development across southern Peru is consistent with the early Miocene onset and middle Miocene intensification of hyperarid conditions along the central Andean Pacific coast.

## Introduction

The central Andean orogen is the archetypal ocean-continent convergent boundary, and Earth’s second largest modern orogenic plateau^1^. The tectonic evolution of the greater Andean orogen, Earth’s longest continuous modern mountain chain, in part controls climate patterns of the region. The central Andes receives the overwhelming majority of its moisture from Atlantic-sourced equatorial easterlies that traverse the Amazon basin^2^. Progressive rainout of precipitation with relatively higher ^2^H (deuterium, D) and ^18^O isotopic concentrations over the Amazon is balanced by re-evaporated (recycled) moisture and forest transpiration within the Amazon basin, resulting in an inland isotopic gradient considerably lower than the global average^3,4^. Moisture is deflected southward by the topography of the northern Andes, forming the South American low-level jet. Moisture-bearing air masses impinge on the central Andes from the east and northeast, resulting in heavy precipitation along the Eastern Cordillera, the majority of which falls in austral summer. Although some studies have suggested the uplift of the Andes did not play a significant role in the development of arid conditions along the central Andean Pacific coast^5^, others have pointed to this orographic barrier as being a dominant driver of hyperarid conditions^6^. Whether uplift of the Andes contributed to arid conditions along the central Andean Pacific coast and the Atacama Desert (one of the most arid regions on Earth) remains a debated topic. Hence, evaluating the timing of surface uplift provides a test of such climate-elevation interactions in the central Andes^7–9^ by determining if the development of this major orographic barrier coincided with the establishment of hyperarid conditions along the Pacific coast.

Development of the 4–6 km-high topography in the central Andes took place in the Cenozoic due to ongoing east-directed subduction of oceanic lithosphere^1^. However, details of the timing, rate, and locus of attainment of high topography have been the subject of considerable debate^9–12^. All geodynamic models explaining generation of extreme elevations in the Andes (i.e., >3 km), irrespective of the controlling mechanism ultimately driving the development of elevated topography, require absence or removal of significantly thickened and/or densified mantle lithosphere in order to generate isostatic adjustment and surface uplift^11^. Differing processes of lithospheric removal predict unique spatial and temporal patterns of surface uplift and crustal shortening. For example, removal of lower crust and/or mantle lithosphere via large-scale mantle convection or foundering of Rayleigh-Taylor instabilities has been proposed as a viable mechanism for wholesale^13^ or localized^14^ rapid (>0.5 km/Myr) surface uplift, respectively. In these models, uplift is largely decoupled from crustal shortening. Alternatively, incremental removal by ablative subduction^15^, thermal weakening^16^, or simple shear underthrusting of lower crust and mantle lithosphere^17^, predicts lower (<0.5 km/Myr) rates of surface uplift in concert with crustal shortening.

Surface uplift is central to understanding the role that tectonics has on climate. The horizontal forces that drive tectonic plate motions are proportional to the forces required to uplift and support mountain ranges^18^; topography in turn drives critical climate feedbacks of local^8^, regional^11^, and global^19^ significance. Despite such interest and importance in the study of vertical motion of the Earth’s surface, it remains difficult to measure in the geologic past because only proxy methods may be used. Methods applied to the central Andes have been based on timing of crustal shortening^16^, or on geomorphic features such as regional tilt and monocline development^20,21^, paleosurface peneplain mapping^22^, and river incision^23^. Alternatively, biological proxies, such as phylogenetics of high-elevation biotaxa^24^ and leaf physiognomy^25^, have been used to infer surface uplift of the central Andes. More recently developed paleoelevation proxies exploit the stable isotopic record by measuring δ^18^O in soil carbonates^9^, mean annual air temperatures inferred from clumped isotope Δ_47_ variations in carbonate^26^, and paleoenvironmental waters preserved in hydrated volcanic glass^27–29^. These stable isotopic proxies may be subject to diagenetic alteration and/or isotopic overprinting; however, when preserved pristinely, these proxies can be coupled with isotopic or temperature lapse rates to infer paleoelevation. Interpretations based on modern lapse rates must account for changes in isotopic composition accompanying global climate change^30^, and potentially for changing lapse rates with increasing surface elevations^31,32^. Paleoelevation proxy materials may be complicated by limited preservation potential, and recording rock uplift rather than surface uplift^18^.

Application of D/H (the ratio of heavy hydrogen, Deuterium, ^2^H, to light hydrogen, ^1^H) isotopic analysis of hydrated volcanic glass to paleoaltimetry is a particularly powerful stable isotopic technique because water-glass isotopic fractionation is independent of temperature, and glass is a durable closed system once fully hydrated^33,34^. There has long been definitive evidence for the existence of both hydroxyl groups and molecular water in silicate glasses^35^ incorporated at low temperatures subsequent to glass quenching rather than from the original magmatic source^33^. Although the primary mechanism of low-temperature water absorption is debated^36^, there is general consensus that incorporation of water into glass involves the selective removal of susceptible alkali ion reaction sites on the glass surface^37^, and continues with subsequent diffusion into glass through replacement of large-radius alkali metal cations (e.g., Na^+^, K^+^) with H^+^ and D^+^ ions^34,38^. Following hydration and uptake into the glass structure, glasses form alteration products in the form of gels, phyllosilicates, or phosphates^36^, a process recognized as early as the 1840s^39^. Formation of gel layers in glasses rich in SiO_2_ and depleted in alkali metal oxides (e.g., Na_2_O, K_2_O) acts to impede further corrosion on the surfaces of glass shards^34^. Insofar as glass is not significantly altered, heated, or subjected to extreme pH conditions^40^, glass D/H compositions are representative of time-averaged (over 1000 s of years) ancient environmental water^33^, and thus is useful in determining past elevations of orogens with extensive volcanism.

Modern water stable isotopic compositions provide a foundation for interpreting past elevations from ancient environmental waters preserved in hydrated volcanic glasses. However, isotopic compositions of moisture in the Peruvian central Andes, and orogenic plateaus in general, can be variable. Although rainout during orographic ascent of moisture along the Eastern Cordillera of southern Peru produces an expected isotopic gradient from the relative depletion of D isotopes that inversely correlates with elevation, as observed globally across the windward sides of mountains^41^, leeward moisture traverses over a high orogenic plateau rather than returning to relatively low elevations (e.g., the Sierra Nevada Mountains). During the traverse across high topography, moisture undergoes subcloud evaporation and recycling effects resulting in an inland gradient to heavier isotopic compositions^42^.

**Figure 1:**
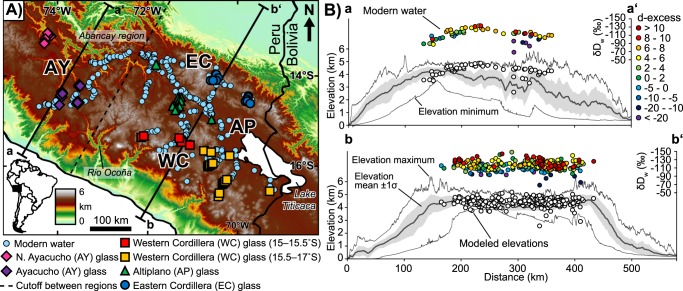
Study area, sample locations, and modern water elevation modeling. (**A**) Digital elevation model showing the northern extent of the Andean plateau generated from Shuttle Radar Topography Mission (SRTM) 90 m elevation data. Physiographic regions characterizing the central Andes: AY = Ayacucho, WC = Western Cordilleran, AP = Altiplano. EC = Eastern Cordillera. Topographic swaths along a–a’ and b–b’ are 140 and 340 km wide, respectively; the dashed line is an arbitrary cutoff between the topographic swaths and modern water data. Modern water data include results from Bershaw^42^ and volcanic samples include results from Saylor and Horton^12^. (**B**) Topographic swaths showing mean, minimum, maximum, and 1σ standard deviation elevation. Modern water measurements are color-coded to d-excess values. White circles are mean catchment elevations calculated from modern waters using a modified non-linear isotopic lapse rate^43^.

We present stable isotopic data from 376 modern stream water samples (179 new, 197 published^42^), and 136 volcanic glass samples (117 new samples, 19 published^12^) to determine surface uplift patterns across the central Andes of southern Peru (Fig. 1A). Results show that mean catchment elevations can be calculated by applying a nonlinear isotopic lapse rate^43^ to modern water D/H ratios. Further, successful reproduction of modern elevations from young (i.e., Pliocene–Pleistocene) glass samples using this same lapse rate sets the foundation for interpreting paleoelevations from preserved waters deeper in the geologic past. Paleoelevation estimates from hydrated volcanic glasses, temporally controlled by new zircon U-Pb dates and published age data, reveal spatially and temporally variable late Cenozoic surface uplift magnitudes and rates across the Peruvian central Andes. Differing surface uplift rates and temporal coincidence with crustal shortening implicate multiple geodynamic processes active during the Cenozoic construction of the Peruvian central Andes^10^. The timing of development of high topography is consistent with early to middle Miocene estimates for the onset of hyperarid conditions along the Pacific coast of the central Andes^6^.

## Results

### Modern stream water

**Figure 2:**
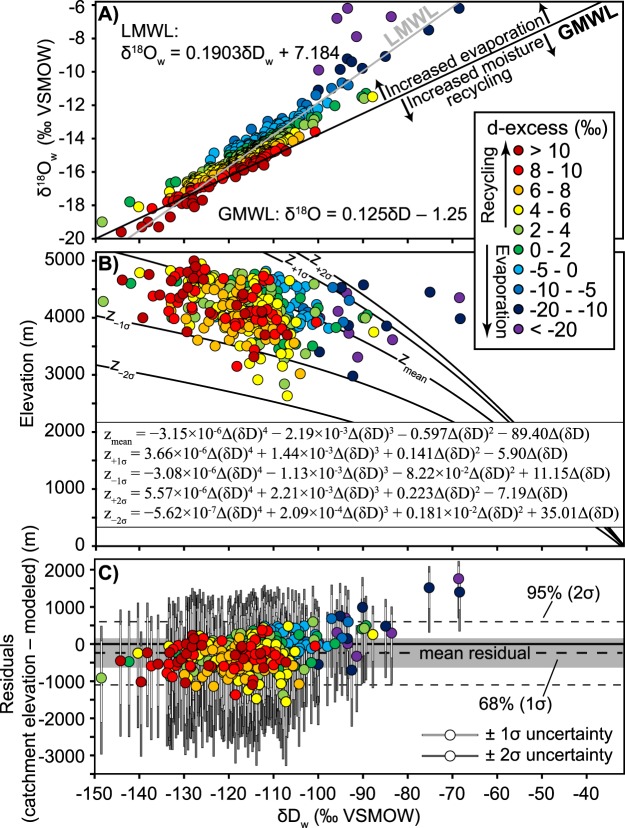
Modern water stable isotope results, lapse rates, and residuals. (**A**) Modern water δD_w_ and δ^18^O_w_ (relative to VSMOW) compared to GMWL and color-coded to d-excess. Negative d-excess values are interpreted to be the result of moisture modified by evaporative effects, whereas positive d-excess values are interpreted to be the result of moisture recycling^45,46^. Results include water data from Bershaw^42^ (**B**) δD_w_ values converted to elevation based on a non-linear isotopic lapse rate modified from Rowley^43^. (**C**) Residuals (mean catchment elevations – modeled elevations); the majority of water values yield overestimates in catchment mean elevation resulting in a negative mean residual (−246 ± 427 m at 1σ).

Modern water oxygen and D/H isotopic compositions, reported in parts per thousand (‰) deviation from Vienna Standard Mean Ocean Water (VSMOW) as δ^18^O and δD, respectively, reveal a deviation from the global meteoric water line^44^ (GMWL, defined as δD = 8 × δ^18^O + 10) (Figs 1B and 2A). Comparison of the local meteoric water line (LMWL) to the GMWL allows for calculation of the δD in individual water samples resulting from non-equilibrium conditions (d-excess = δD − 8 × δ^18^O), which can be used to understand evaporative processes and/or moisture sourcing^45,46^. The LMWL deviates from the GMWL primarily due to evaporative effects rather than moisture recycling, as the very negative d-excess values largely control the regression of the LMWL (Fig. 2A).

Modern water D/H isotopic compositions were converted to mean catchment elevation values using a non-linear isotopic lapse rate^43^ (Fig. 2B). This lapse rate was derived from a polynomial regression of modeled changes in the O isotopic composition of precipitation due to adiabatic expansion and condensation with changes in elevation^43^. Specifically, the model implements combinations of modern temperature and relative humidity which result in δ^18^O in precipitation values of between 0 and −25‰^43^. The lapse rate and associated uncertainties were linearly converted from the O system to D/H (δD) using the GMWL (Fig. 2B and Supplementary Information). The lapse rate is benchmarked to low-elevation isotopic compositions and applied to a specific region by calculating the hypsometric mean elevation for incremental changes (ΔδD) from that low-elevation baseline^43^ (Fig. 2B). Here, we normalize to δD = −31.6‰, converted using the GMWL from the average isotopic composition of precipitation reported for Trinidad, Bolivia of δ^18^O = −5.2‰^43^.

Despite significant moisture recycling, variably arid conditions, and evaporative effects resulting in divergence of the LMWL from the GMWL (Fig. 2A), the modeled modern water isotopic compositions reproduce elevation estimates with reasonable error compared to water sample mean hypsometric elevations. Results show that 96% of absolute residuals (|catchment mean elevation – modeled elevation|) are less than 1000 m; 71% are less than 500 m (Fig. 2C). The lapse rate performs better to the south (b–b’, Fig. 1) with 98% and 72% of absolute residuals less than 1000 m and 500 m, compared to 86% and 66% in the north, respectively. Modeled elevations yield minor elevation overestimation (300–400 m) along the Eastern Cordillera in southern Peru due to a higher isotopic gradient than that of the non-linear isotopic lapse rate. This effect is balanced by increased moisture recycling and a gradual shift to more positive δD and δ^18^O values to the west^42^, resulting in the lowest residuals in the Altiplano, and minor elevation underestimates in the Western Cordillera (10 s of m) and Ayacucho region (100–200 m). For a more in-depth discussion on spatial patterns of δD_w_ across southern Peru see Bershaw *et al*.^42^. Collectively, reconstructed elevations yield an average overestimate compared to true elevations with a mean residual of −246 ± 427 m at 1σ (±854 m at 2σ). There is little to no correlation between d-excess and residual values, except for highly evaporated waters (d-excess <−10‰). Thus, barring extreme evaporative effects, elevation estimates based on δD values are applicable even in arid regions assuming selection of an appropriate lapse rate.

### Ancient water preserved in hydrated volcanic glass

**Figure 3:**
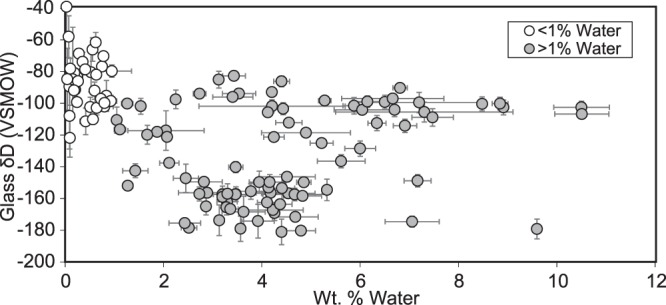
Volcanic glass weight % water plotted against uncorrected δD_g_ measurements. Glasses with low hydration (<1 weight %) are biased toward positive values and are not considered in paleoelevation calculations.

Volcanic glass stable isotopic results (δD_g_) are considered in four groups based on physiographic region: Ayacucho, Western Cordillera, Altiplano, and Eastern Cordillera (Fig. 1A). Glasses that are incompletely hydrated^33^ (<1 weight % water) are biased toward relatively positive δD values (δD_g_ = −100 to −40‰) (Fig. 3). These specious results are likely due to sensitivity limitations during isotopic measurement, and so were excluded from further consideration. Glasses hydrated with >1 weight % water yielded a range of δD_g_ values from −181 to −83‰ and no correlation with weight % water (Fig. 3). Consideration of volcanic glass δD_g_ from non-hydrated glasses (<1 weight % water) could lead to geologic misinterpretation, as these data tend to yield systematically more positive (−100 to −40‰) δD_g_ values resulting in surface uplift underestimates and/or larger shifts in isotopic records than may have taken place (Fig. 3).

**Figure 4:**
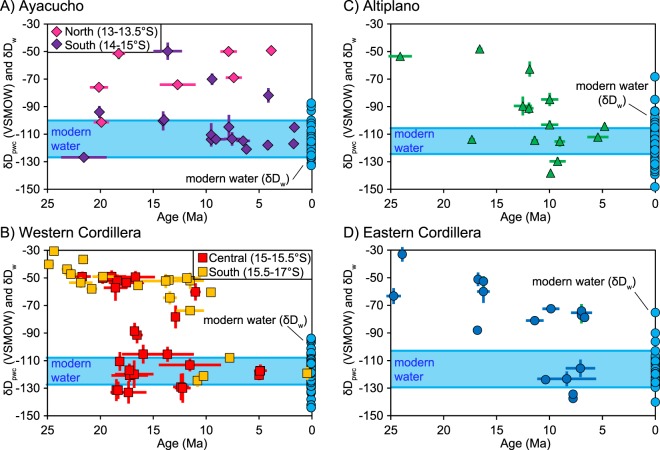
Comparison of volcanic glass paleowater corrected for Oligocene – modern cooling (δD_pwc_) and modern water stable isotopic compositions. (**A**) Ayacucho region; (**B**) Western Cordillera (including data from Saylor and Horton^12^), note the results are separated into central (15°S–15.5°S) and southern (15.5°S–17°S) regions; (**C**) Altiplano; and (**D**) Eastern Cordillera. Volcanic glass δD_g_ results are corrected for water-glass fractionation based on Friedman *et al*.^33^. Blue circles are modern waters for each physiographic region. Symbols are keyed to Fig. 1A.

Ancient environmental water isotopic compositions (paleowater, δD_pw_) were determined by correcting for water–glass hydrogen isotope fractionation^33^ (δD_pw_ = 1.034 × (1000 + δD_g_) − 1000). All δD_pw_ values were also corrected for global cooling and relative increase in global water δ^18^O and δD since the late Oligocene^30^ (δD_pwc)_ (see Supplementary Information). Forgoing the correction from δD_pw_ to δD_pwc_ results in a trend toward more positive isotopic compositions through time, which could erroneously be interpreted as a decrease in elevation or increase in aridity (see Supplementary Information). Because ancient waters were subject to the same evaporative effects as in the modern, interpretation of isotopic changes in moisture is based on the most negative δD_pwc_ values for each physiographic region^42^; this also assumes we have indeed sampled the earliest volcanic rocks recording the isotopic shift to modern D/H and thus yields a youngest estimate for each region. The youngest δD_pwc_ (<10 Ma) are similar to modern water isotopic values in each physiographic region (Fig. 4). The Ayacucho region shows near modern values as early as ~22 Ma, with a divergence in later isotopic trends; samples from northern Ayacucho (13–13.5°S) reveal a steady increase to more positive δD_pwc_ values from 20–4 Ma, whereas southern Ayacucho (14–15°S) maintains near modern values to the present (Fig. 4A). δD_pwc_ from the Western Cordillera, Altiplano, and Eastern Cordillera show spatially and temporally variable shifts to modern values (Fig. 4). The Western Cordillera shows two separate shifts to modern δD_pwc_ that differ based on latitude. Results from the central Western Cordillera between 15°S to 15.5°S give values of ~−50‰ at 22–17 Ma that transition to modern values by 17 Ma (Fig. 4B). Southern Western Cordilleran volcanic glasses between 15.5°S and 17°S yield values of −40 to −30‰ at 25–22 Ma that first shifts from ~−60 to ~−75‰ between 21 and 15 Ma then to modern values between 15 and 10 Ma (Fig. 4B). δD_pwc_ values from the Altiplano yield potential shifts from ~−75‰ to modern values at either ~17 Ma or between 15 and 10 Ma (Fig. 4C). The Eastern Cordillera also shows a slower transition of δD_pwc_ with values of ~−85‰ at ~25 Ma that steadily decrease to modern water δD_w_ values by 10 Ma (Fig. 4D).

### Paleoelevation calculations

**Figure 5:**
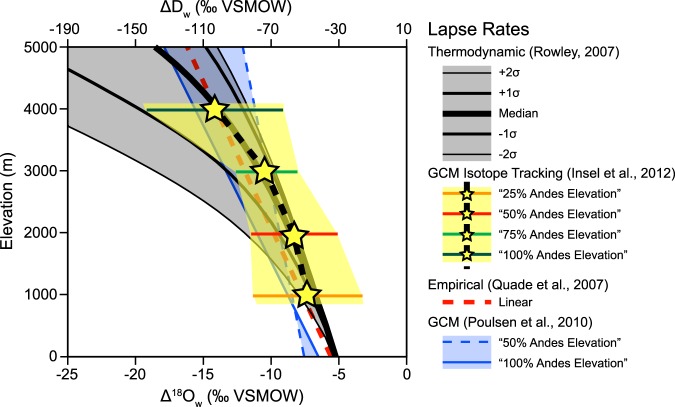
Isotopic lapse rates used to calculate paleoelevation and surface uplift magnitude. Lapse rates include: the thermodynamically-derived non-linear model from Rowley^43^; isotope-tracking general circulation model (GCM) with 25, 50, 75, and 100% of Andean elevations from Insel *et al*.^32^; linear empirical from Quade *et al*.^47^; and GCM with 50 and 100% Andean elevation from Poulsen *et al*.^31^. Note the similarity in the Insel *et al*.^32^ and Rowley^43^ best-fit lapse rates.

**Figure 6:**
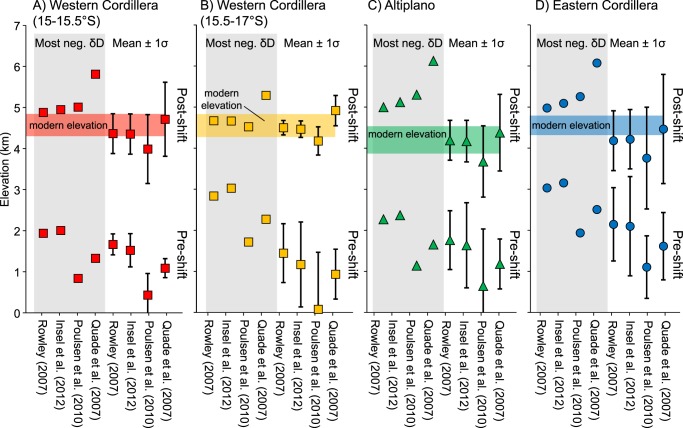
Surface uplift estimates for each physiographic region based on lapse rates in Fig. 5. Estimates are calculated based on the most negative and mean pre-shift (>−90‰) and post-shift (<−90‰) water-glass fractionation corrected dD_pw_ values^33^. Estimates take into account Oligocene – Pleistocene global cooling climate correction (see text and Supplementary Information for details). Shaded horizontal colored bars are ±1σ standard deviation of modern elevation.

Volcanic glass isotopic compositions require vertical lapse rates in order to convert the δD_pwc_ values into paleoelevations. We consider four different isotopic lapse rates (Fig. 5): (1) the thermodynamically-derived non-linear lapse rate^43^ discussed above; (2) a non-linear lapse rate derived from an isotope-enabled atmospheric general circulation model (GCM) that tracks oxygen isotope vapor with changes to Andean elevation to 25%, 50%, 75% and 100% of modern elevation^32^; (3) a linear lapse rate derived from GCM results that shows a threshold change for Andean elevations of 50% and modern^31^; and (4) an empirically-derived, 2.1‰/km linear lapse rate^47^, similar to the 2.8‰/km decrease in δ^18^O observed globally (on average) along the windward side of orogens^41^. Surface uplift magnitudes calculated based on the most negative and mean pre-shift (>−90‰) and post-shift (<−90‰) δD_pw_ values using the four different lapse rates above are summarized in Fig. 6. All lapse rates tend to overestimate modern surface elevations when Oligocene – Pleistocene global cooling and progressive shift toward isotopically positive δD is not taken into account (Supplementary Information). Results based on linear isotopic lapse rates^31,47^ yield surface uplift magnitudes that are 500–1500 m greater than estimates calculated using non-linear lapse rates^32,43^, and in many cases gross overestimates of modern elevation (Fig. 6 and Supplementary Information). Surface uplift calculations based climate corrected δD_pwc_ values and non-linear isotopic lapse rates^32,43^ are the closest on average to modern elevations (Fig. 6 and Supplementary Information). Further, the non-linear isotopic lapse rates yield very similar results for all pre- and post-shift isotopic values, which is unsurprising given their best-fit lapse rates are nearly identical (Fig. 5). Therefore, surface uplift estimates presented below are based on the non-linear thermodynamic model discussed above^43^ (Fig. 5); estimates based on the other three lapse rates, with and without a climate correction for global cooling since the Oligocene (δD_pw_ and δD_pwc_) are presented in the Supplementary Information.

**Figure 7:**
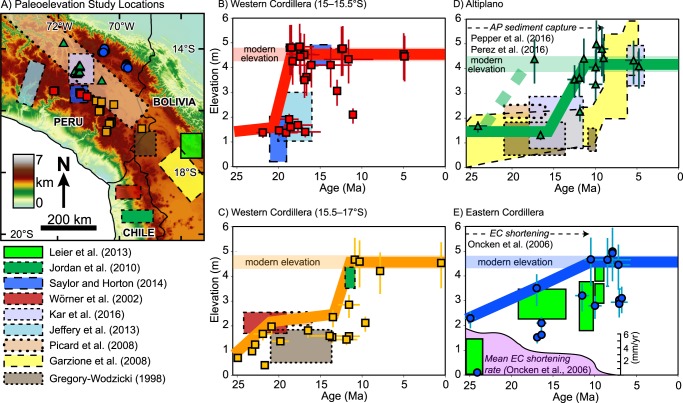
Paleoelevation results compared to published paleoelevation estimates and ancillary geological data. (**A**) Locations for this and previous studies; for the latter only absolute elevation estimates are included. Results reveal variable surface uplift records consistent with previous studies for the (**B**) central Western Cordillera (15°S–15.5°S), (**C**) southern Western Cordillera (15.5°S–17°S), (**D**) Altiplano, and (**E**) Eastern Cordillera. Paleoelevations were calculated based on the thermodynamic lapse rate^43^ and incorporate a climate correction that accounts for Oligocene – Pleistocene global cooling. Youngest low elevation age for Kar *et al*.^48^ calculated assuming a linear sedimentation rate for the Descanso Formation. Shaded horizontal colored bars in parts B-E are ±1σ standard deviation of modern elevation.

Regardless of the lapse rate used to calculate past elevations, results reveal spatial and temporal variability in surface uplift patterns across the Peruvian central Andes. The Ayacucho region was at or above modern elevation as early as ~22 Ma (Fig. 4A). The steady increase in δD_pwc_ values in northern Ayacucho could be interpreted as a steady decrease followed by a late (post 4 Ma) increase in paleoelevation; however, the data in this region are sparse, and may have recorded evaporatively enriched waters, as values in the southern Ayacucho region are consistently similar to the modern (Fig. 4A). The Western Cordillera shows variation along orogenic strike: at 15°S–15.5°S elevations increased from 1.5–2.0 km to modern (~4.5 km) between 22 and 17 Ma at (Fig. 7B); at 15.5–17°S elevation changes from 1.0 to 2.0 km between 25 and 20 Ma, followed by an increase to modern elevation (~4.5 km) between 15 and 10 Ma (Fig. 7C). The Altiplano was uplifted slightly slower and later, increasing from ~1.5 km to modern elevation (~4 km) either between 24 and 17 Ma, or 15 and 10 Ma (Fig. 7D). The Eastern Cordillera shows protracted surface uplift from ~2.5 m at ~25 Ma to modern elevations (~4.5 km) by 10 Ma (Fig. 7E).

## Discussion

**Figure 8:**
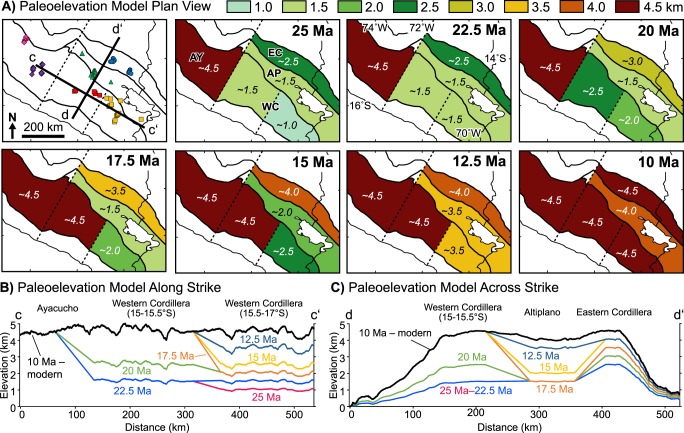
Paleoelevation model showing variable surface uplift history of the Peruvian central Andes at resolution of general circulation models in (**A**) map view and cross section (topographic profile) view (**B**) along strike (c–c’) and (**C**) across strike (d–d’). Topographic profiles were calculated by multiplying the modern mean topography by percentage elevation through time with boundaries linearly interpolated between physiographic regions. Dashed lines are arbitrarily drawn to separate the Ayacucho region and the central and southern Western Cordillera.

Stable isotopic results from hydrated volcanic glass reveal spatially and temporally variable surface uplift estimates across the Peruvian central Andes that are consistent with previous paleoelevation studies (Figs 7 and 8). Early, high topography in the Ayacucho region by ~22 Ma is consistent with middle Miocene to modern Rio Ocoña incision modeling of the central to northern Western Cordillera (Fig. 7B) that indicates elevations of 1–3 km by 20–16 Ma^23^. Our new paleoelevation estimates for the central Western Cordillera are the most robust for any region in southern Peru and are consistent with previous paleoelevation estimates based on hydrated volcanic glass that indicate rapid early Miocene surface uplift^12^. Results for the southern Western Cordillera and northern Altiplano are consistent with paleoelevation estimates from leaf physiognomy suggesting these regions attained no more than half their modern day elevations by 25–18 Ma^25^. These results are within uncertainty of elevation estimates based on molecular phylogenetics and phylochronologic analysis that require at least 2 km elevation in the early Miocene in southernmost Peru in order to sustain high-elevation-dwelling *Globodera pallida* potato parasite nematodes^24^. The southern Western Cordillera estimates are also consistent with paleoelevation estimates based on deformation of large-volume ignimbrite deposits that suggest surface uplift started as early as 23 Ma^48^; regional tilting and monocline development suggesting relative elevation increase of 2 km between 26 and 8 Ma^49^ and absolute elevations of 1.7–2.5 km since 19 Ma^21^ and ~3800 km by 11 Ma^20^; and modeling of cosmogenic ^3^He accumulation in alluvial boulders indicating attainment of significant elevation prior to 14 Ma^50^. Results suggest surface uplift and attainment of modern elevations in the northern Altiplano occurred earlier than previous estimates of 10–6 Ma based on δ^18^O stable isotopes in carbonates, Δ_47_ clumped isotopes, pollen assemblages, and leaf wax lipids^51^. Although results from Kar *et al*.^51^ preclude early elevation gain between 25 and 17 Ma, they do not preclude surface uplift between 15 and 10 Ma. In fact, our results effectively fill in the gaps between their early-middle Miocene low-elevation and late Miocene high-elevation estimates (Fig. 7D), which occur between their 1400 and 1700 m stratigraphic interval between 18.7 and 5 Ma^51^. Although limited compared to other regions, paleoelevation estimates in the Eastern Cordillera are consistent with paleoelevation estimates from O and C isotopic data from the northern Bolivian central Andes that suggest early (>24 Ma) low elevation followed by stepwise elevation gain to ~2.5 km between 20 and 15 Ma, then to modern elevation <10 Ma^52^.

New surface uplift estimates across the Peruvian central Andes are consistent with independent geologic observations. For example, in the northernmost Altiplano near Cusco, Peru sediments were consistently sourced from the Western Cordillera throughout the Cenozoic^53^, which suggests a relatively low Altiplano. To the south, west-directed paleocurrents sourcing Eastern Cordilleran sediments from 30–20 Ma followed by a 17 Ma switch to east-directed paleocurrents sourcing western sediments in the Ayaviri region^54^ is consistent with a relatively higher elevation Eastern Cordillera compared to the Altiplano (Fig. 8A,C). Detrital zircon data from Subandean strata and modern rivers east of the Eastern Cordillera contain very few Cenozoic ages^55,56^, which points to sediment capturing within the orogenic hinterland due to blocking by a high Eastern Cordillera and/or sediment accumulation in a relatively low Altiplano (Fig. 8C). Middle to late Miocene unconformity development followed by development of isolated intermontane basins in the northern Altiplano^53,57^ provides support for the timing of rapid surface uplift in the northernmost Altiplano between 15 and 10 Ma (Fig. 7C). The Tincopalca basin in the southern Western Cordillera shows a lithofacies transition from fluvial-lacustrine to evaporitic that coincides with interpreted timing of surface uplift^57^. Finally, the timing of surface uplift in the Eastern Cordillera is consistent with the late Paleogene onset of shortening which peaked at ~25 Ma and then steadily decreased until its cessation at 10 Ma^58^.

Spatial and temporal variability in surface uplift rate requires different geodynamic processes to generate and maintain high topography. Because no elevation change is recorded in the Ayacucho region, and it may have been at high elevation earlier than ~22 Ma, we cannot infer any geodynamic process that controlled the development of high topography. Although there are likely no sharp boundaries between the north, central, and southern Western Cordillera (dashed lines in Figs 1A and 8A), the modern lithospheric structure of this northern region is considerably different. The mantle lithosphere is thinner and subduction angle is steeper in the south compared to the north where there is a transition between flat and normal slab subduction^59^. Furthermore, the thermal structure is different farther south along strike, as there are low velocity zones directly beneath the central and southern Western Cordillera at 80–105 km depth^59^ identified by S wave velocity structure of the upper mantle from joint inversion of ambient noise and earthquake-generated surface waves^59^. High surface uplift rates (>0.5 km/Myr) of the Western Cordillera and Altiplano are consistent with foundering of mantle lithosphere via Rayleigh-Taylor instabilities. The magnitude of surface uplift in the central Western Cordillera is greater than all other physiographic regions, and the timing precedes that of southern Western Cordillera and Altiplano by at least 5 Myr, which is consistent with accumulation, emplacement, and subsequent foundering of dense materials such as restite, cumulates, and eclogite directly under this part of the thickened magmatic arc. Surface uplift and crustal thickening of the Altiplano may have been driven in part by lower crustal flow from the Western Cordillera or the Eastern Cordillera^60^. The slow surface uplift rate (~0.1 km/Myr) and attainment of elevations >3 km in the Eastern Cordillera, along with higher initial elevations compared to areas in the west, are consistent with crustal shortening in the absence of significantly thickened and/or densified mantle lithosphere due to continual removal via ablative subduction^15^, thermal weakening^16^, or simple-shear underthrusting^17^; we prefer the latter interpretation (see below).

There are a limited number of alternative mechanisms to explain the observed isotopic shifts in lieu of significant changes in elevation. For example, the isotopic shift observed in the Western Cordillera could be driven in part by rain shadow development during eastward building and surface uplift of the Eastern Cordillera, but would likely not produce enough of the observed depletion in δD_pwc_ if moisture returned to lower elevations while traversing a low-elevation Altiplano^9,24,25,51^. An alternative explanation to resolve the surface uplift in the Altiplano region involving sediment infilling would not be possible due to isostatic effects induced by the weight of the sediment itself. A simple calculation shows that 3 km of sediment at 2.6 g/cm^3^ (average for quartz) and mantle density of 3.3 g/cm^3^ would result in only 0.6 km of surface elevation increase; it would require a 12 km thick package of sediment to increase the elevation 2.5 km in the Altiplano basin in the absence of lithospheric removal, which itself is an underestimate because this simple calculation does not take into account sediment compaction. Furthermore, the thickest packages of Neogene strata documented in the Altiplano are only ~5 km^61^; the thickest documented stratigraphic section in the northernmost Altiplano is ~6 km^53^, and was deposited over a much longer timescale (nearly the entire Cenozoic). For the Eastern Cordillera some have proposed an Oligocene – Miocene accumulation and subsequent removal of lower crust and mantle lithosphere via foundering^52^. However, recent ambient noise and surface wave tomography shows a slow anomaly beneath the Subandes that is interpreted to be anisotropic Brazilian craton^59^, which is consistent with simple-shear underthrusting of mantle lithosphere^17^ and the protracted surface uplift rates documented above (Fig. 7E).

Surface uplift of the central Andes resulted in development of a significant orographic barrier to the arid western Pacific coast of central South America. Development of moderately high topography (at least 2 km) in the central Andean orogen between 13°S and 17°S by 15 Ma may have promoted synchronous aridification of the Pacific Andean coast^6,62^. The paleoelevation model presented above predicts that arid conditions along the Pacific coast were first enhanced by early Miocene moderate topography of the Eastern Cordillera, then pushed further into hyperarid conditions following the rapid rise of the Western Cordillera and Altiplano and continued increase of the Eastern Cordillera. This is consistent with long-term erosion rates suggesting a transition from early Miocene arid-semiarid conditions to middle Miocene hyperarid conditions in the Atacama Desert region and southern Peruvian Pacific coast^62^, and middle Miocene orography-induced preservation of hypararid salic gypsisols^8^. However, the relative role of elevation increase in driving hyperaridity is uncertain. Some studies have suggested the Andes are not the cause of aridity along the central Andean coast^5^. Specifically, arid conditions along the Pacific coast already result from a combination of its subtropical latitudes, circulation of cold waters from the southeast Pacific Ocean by the Humboldt current, and constant high atmospheric pressure conditions, all of which act to drive away atmospheric moisture^62,63^. Furthermore, general circulation modeling shows that the mere presence of the Andes, regardless of its elevation, is enough to lower sea surface temperature and promote evaporation in the region^63^. The coincidental timing of attainment of extreme elevations and independently determined timing of onset of hyperaridity suggest hyperaridity may be combination of latitude, ocean currents, pressure, and orographic development. Specifically, if arid conditions existed prior to the development of the high elevation central Andes, then surface uplift of the Peruvian central Andes pushed the already arid Pacific coast into the deep rain shadow in which it sits today^6^, triggering the establishment of hyperarid conditions that have persisted until the modern.

The role of elevation increase on regional climate and isotopic lapse rates remains an open question that may be better understood with independent models of landscape evolution. For example, paleoclimate models predict increasingly wetter conditions and concomitant isotopic decreases due to the amount effect during the transition to high elevations^31,32^. The details of the interplay between climate and elevation, particularly those associated with changes in isotopic lapse rates^11,31,32^, are testable with the paleoelevation model presented here, as model results are at the resolution of general circulation models (Fig. 8A). To date, the effects of spatially variable surface uplift of the Andes on regional climate have not been considered. Rather, research utilizing GCMs to assess the effect of Andean uplift on regional climate have only considered wholesale changes in elevation with the modern configuration of the Andes^11,31,32^. Variable surface uplift patterns could have profound effects on the local and regional climate, which in turn would effect changes in the stable isotopic record. For example, the emerging Eastern Cordillera may have been high enough (~2.5 km) to block moisture-laden air masses as early as 25 Ma (Fig. 5). If the Altiplano was as low as predicted before 15 Ma (~1.5 km), and so too was the Abancay region east of Ayacucho to the north^23,24^, then moisture would have penetrated far into the orogenic hinterland resulting in arid, but not hyperarid conditions along the central Andean Pacific coast, which is consistent with the observed onset of hyperaridity after ~17 Ma^62^. Results of this study documenting variable surface uplift both along- and across-strike provide a new avenue of research focused on tectonic-climate interaction.

## Materials and Methods

Stable isotopic data are reported for 376 modern stream water samples (179 new) and 136 volcanic glass samples (117 new samples, 488 individual aliquots) spanning the central Andes of southern Peru (Fig. 1A). New water samples were collected during two field seasons in the austral winters of 2015 and 2016, and analyzed at the University of Rochester Stable Isotope Ratios in the Environment Analytical Laboratory (SIREAL) using off-axis integrated cavity output spectroscopy on a Los Gatos Research liquid water isotope analyzer (detailed results are reported in Supplementary Information). For each analysis, ~900 nL of water were injected into a heated block of the water isotope analyzer; a single analysis is based on fourteen individual injections, ignoring the first three in order to avoid memory effects, with a minimum of five injections used to calculate the final isotopic value. Results are reported relative to VSMOW and normalized such that H and O are both 0‰ for VSMOW, and –428 and –55.5 for Vienna Standard Light Antarctic Precipitation (VSLAP). Volcanic samples were crushed and milled to <500 μm and water tabled. Glass was prepared following recently-documented protocols^27^. The light water table fraction was wet sieved to between 125 and 250 um, soaked in 6 N HCl for 24 hours, sonic bathed for 1 to 4 hours, dried, magnetically separated, density separated using lithium sodium metatungstate (LST) with a density between 2.40 and 2.45 g/mL, and bathed in ~10% HF for 8 to 10 s. Samples were examined with a petrographic microscope to check for purity. Aliquots of 1.5–2.5 mg were packed into Ag crucibles and analyzed on a thermal combustion element analyzer at the University of Texas at Austin and the University of Arizona. For the 45 samples lacking age control, zircons were extracted from the water table heavy fraction using standard heavy liquid separation with methylene iodide with a density between 3.30 and 3.32 g/cm^3^ and magnetic separation. Zircons were analyzed by laser-ablation inductively-coupled-plasma mass-spectrometry (LA-ICP-MS) at the University of Houston and the LaserChron center at the University of Arizona (see Supplementary Information for details of LA-ICP-MS, data reduction, and initial-Pb corrections).

## Supplementary information

Supplementary Information

S1

S2

S3
