# Supplementary material for: Implications of variable late Cenozoic surface uplift across the Peruvian central Andes
Source: Sci Rep. 2019 Mar 19;9:4877. doi: 10.1038/s41598-019-41257-3 (PMC6424981; doi:10.1038/s41598-019-41257-3)
Supplement: Supplementary file 1 — Supplementary Information [file 41598_2019_41257_MOESM1_ESM.pdf]

Nature Scientific Reports

Supplementary Information for

Implications of variable late Cenozoic surface uplift across the Peruvian central Andes

Kurt E. Sundell[1,2†], Joel E. Saylor[1], Thomas J. Lapen[1], Brian K. Horton[3]

[1]Department of Earth and Atmospheric Sciences, University of Houston, Houston, Texas

[2]Now at Department of Geosciences, University of Arizona, Tucson, AZ 85721

[3]Department of Geological Sciences and Institute for Geophysics, University of Texas at Austin, Austin, TX 78712

[†]Corresponding author: sundell@email.arizona.edu

Contents of this file

Text S1: Detrital zircon U-Pb geochronology methods and raw data

Text S2: Conversion of lapse rate from Rowley (2007) from O to H

Figure S1: Example $\delta^{18}O$ data vs mean catchment hypsometry

Figure S2: Example $\delta^{18}O$ data converted to δD with GMWL

Figure S3: Correction for global changes in ocean water chemistry based on Zachos et al. (2001)

Figure S4: Surface uplift estimates for pre- and post-shift δD_{pw}

Figure S5: Surface uplift summary (without climate correction) for pre- and post-shift δD_{pw}

Figure S6. Paleoelevation for each region based on Rowley (2007) lapse rate

Figure S7. Paleoelevation for each region based on Insel et al. (2012)

Figure S8. Paleoelevation for each region based on Poulsen et al. (2010) "INT" lapse rate

Figure S9. Paleoelevation for each region based on Poulsen et al. (2010) "MOD" lapse rate

Figure S10. Paleoelevation for each region based on Quade et al. (2007) lapse rate

Table S1. Surface uplift magnitudes summary

Additional Supporting Information (Data files uploaded separately)

Data File S1: Volcanic glass stable isotopic results

Data Set S2: Detrital zircon U-Pb data and age calculations (2σ uncertainties)

Data Set S3: Modern water data and elevation modeling

Introduction

This supporting information provides a description of the methods used to generate the detrital zircon U-Pb data and alternative calculations based on different lapse rates and forgoing the climate correction to the volcanic glass stable isotopic data.

Text S1. University of Houston zircon U-Pb geochronology procedures, analysis, and data reduction

Zircon grains were separated from volcanic rock samples following standard mineral separation procedures of crushing, disc milling, water tabling, heavy liquids and magnetic separation. Euhedral zircon grains were picked and placed onto two-sided tape and/or mounted in epoxy and polished for analysis. Grains were ablated using a Photon Machines Analyte 193 ArF excimer laser attached to a pulse counting detector fitted to a Varian 810 quadrupole mass spectrometer (Shaulis et al., 2010). For all analyses a 20 – 40 µm spot size (depending on sample grain size yield) was used with a fluence of 2.99 J/cm2 and 10 Hz repetition rate for 200 – 300 shots, resulting in approximately 20 – 30 seconds of ablate time, with 15 – 20 seconds of background measurement and 10 – 15 seconds of washout following each analysis. All other machine parameters are similar to those outlined in Shaulis et al. (2010).

ICP-MS data was recorded and exported using Quantum. Raw data was baseline corrected and integrated using an in-house MATLAB-based graphical user interface (GUI) at the University of Houston, UPbToolbox (Sundell, 2017). Individual analyses were background corrected by taking the mean counts per second for each isotope for the first ~12 seconds and subtracting that value from each integrated spectrum for the total analysis time. A constant integration window was chosen for each sample run (15 – 25 seconds, 3 – 4 seconds after start of ablation) in order to calculate mean isotopic ratios and 2 standard error for each integration. Integration windows were held constant for all analyses and standards for individual runs because no downhole fractionation correction was conducted (c.f., Košler et al., 2002). This 'averaging' approach was used to calculate raw standard ratios for fractionation and drift corrections.

Results were filtered based on percent sample uncertainty and interpreted detrital grains. In lieu of making a common Pb correction (Stacey & Kramers, 1975), we corrected samples showing significant common Pb by calculating the lower intercept of a Tera-Wasserburg concordia plot. All other reported ages are weighted mean $^{206}Pb/^{238}U$ age dates. Analyses with >30% $^{206}Pb/^{238}U$ uncertainty were not considered in age calculations, nor were ages significantly older than the primary young age mode; this latter data filtering is required due to the presence of detrital grains observed in many of the volcanic outcrops in the field and in hand sample. All

analyses (accepted and rejected), as well as standard analyses for each analytical session, are reported in Supplementary Data Set S2.

We used well-characterized primary and secondary standard reference materials. Plešovice zircon, originating from potassic granulite in the southern Bohemian Massif, Czech Republic, with an ID-TIMS age of 337.13 ± 0.37 Ma (Sláma et al., 2008) was used as our primary standard to correct raw mass spectrometer ratios. FC5z zircon from the Duluth Complex in Minnesota, USA, with an accepted age similar to samples AS3 and FC1 from Paces & Miller, (1993) that have an accepted age of 1099.1 ± 0.5 Ma, was used as our external standard to ensure machine run stability and for comparison to primary standards.

References

Košler, J., Fonneland, H., Sylvester, P., Tubrett, M., & Pedersen, R. B. (2002). U–Pb dating of detrital zircons for sediment provenance studies—a comparison of laser ablation ICPMS and SIMS techniques. Chemical Geology, 182(2-4), 605-618.

Paces, J. B., & Miller Jr, J. D. (1993). Precise U-Pb ages of Duluth complex and related mafic intrusions, northeastern Minnesota: Geochronological insights to physical, petrogenetic, paleomagnetic, and tectonomagmatic processes associated with the 1.1 Ga midcontinent rift system. Journal of Geophysical Research: Solid Earth, 98(B8), 13997-14013.

Shaulis, B., Lapen, T. J., & Toms, A. (2010). Signal linearity of an extended range pulse counting detector: Applications to accurate and precise U-Pb dating of zircon by laser ablation quadrupole ICP-MS. Geochemistry, Geophysics, Geosystems, 11(11).

Sláma, J., Košler, J., Condon, D. J., Crowley, J. L., Gerdes, A., Hanchar, J. M., ... & Schaltegger, U. (2008). Plešovice zircon—a new natural reference material for U–Pb and Hf isotopic microanalysis. Chemical Geology, 249(1-2), 1-35.

Stacey, J. T., & Kramers, 1. (1975). Approximation of terrestrial lead isotope evolution by a two-stage model. Earth and planetary science letters, 26(2), 207-221.

Sundell, K. E. (2017). Cenozoic Surface Uplift and Basin Formation in the Peruvian Central Andes (Doctoral dissertation).

Text S2. Conversion of lapse rate from Rowley (2007) from O to H

Thermodynamically-derived lapse rates for calculating hypsometric mean elevations are taken from Rowley (2007) (his Eq 5, based on the $\delta^{18}O$ system wherein

$$z_{mean} = -0.0129\Delta(\delta^{18}O)^4 - 1.121\Delta(\delta^{18}O)^3 - 38.214\Delta(\delta^{18}O)^2 - 715.22\Delta(\delta^{18}O) \qquad \text{Eq. 1}$$

subject to uncertainties of

$$z_{+1\sigma} = 0.0150\Delta(\delta^{18}O)^4 + 0.738\Delta(\delta^{18}O)^3 + 9.031\Delta(\delta^{18}O)^2 - 47.186\Delta(\delta^{18}O) \qquad \text{Eq. 2}$$

$$z_{-1\sigma} = -0.0126\Delta(\delta^{18}O)^4 - 0.580\Delta(\delta^{18}O)^3 - 5.262\Delta(\delta^{18}O)^2 + 89.212\Delta(\delta^{18}O) \qquad \text{Eq. 3}$$

$$z_{+2\sigma} = 0.0228\Delta(\delta^{18}O)^4 + 1.132\Delta(\delta^{18}O)^3 + 14.276\Delta(\delta^{18}O)^2 - 57.547\Delta(\delta^{18}O) \qquad \text{Eq. 4}$$

$$z_{-2\sigma} = -0.0023\Delta(\delta^{18}O)^4 + 0.107\Delta(\delta^{18}O)^3 + 11.611\Delta(\delta^{18}O)^2 + 280.09\Delta(\delta^{18}O) \qquad \text{Eq. 5}$$

where z is the drainage basin hypsometry and Δ refers to the difference between the isotopic composition at sea level and the elevated moisture source where

$$\Delta(\delta^{18}O) = \delta^{18}O_{measured} - \delta^{18}O_{moisture\ source} \qquad \text{Eq. 6}$$

We use $\Delta = -5.2$‰, the weighted average isotopic composition of precipitation at Trinidad, Bolivia. In order to convert this lapse rate from $\delta^{18}O$ to δD we apply a linear transformation using the global meteoric water line (GMWL) from Craig (1961)

$$\delta D = 8\times\delta^{18}O + 10 \qquad \text{Eq. 7}$$

which converts to

$$\delta^{18}O = (\delta D - 10)/8 \qquad \text{Eq. 8}$$

$$\Delta(\delta^{18}O) = [(\delta D - 10)/8]_{measured} - [(\delta D - 10)/8]_{moisture\ source} \qquad \text{Eq. 9}$$

$$\Delta(\delta^{18}O) = (\delta D_{measured} - \delta D_{moisture\ source})/8 \qquad \text{Eq. 10}$$

if

$$\Delta(\delta D) = \delta D_{measured} - \delta D_{moisture\ source} \qquad \text{Eq. 11}$$

then

$$\Delta(\delta^{18}O) = \Delta(\delta D)/8 \qquad \text{Eq. 12}$$

and

$$z_{mean} = -0.0129(\Delta(\delta D)/8)^4 - 1.121(\Delta(\delta D)/8)^3 - 38.214(\Delta(\delta D)/8)^2 - 715.22(\Delta(\delta D)/8) \qquad \text{Eq. 13}$$

$$z_{+1\sigma} = 0.0150(\Delta(\delta D)/8)^4 + 0.738(\Delta(\delta D)/8)^3 + 9.031(\Delta(\delta D)/8)^2 - 47.186(\Delta(\delta D)/8) \quad \text{Eq.14}$$

$$z_{-1\sigma} = -0.0126(\Delta(\delta D)/8)^4 - 0.580(\Delta(\delta D)/8)^3 - 5.262(\Delta(\delta D)/8)^2 + 89.212(\Delta(\delta D)/8) \quad \text{Eq. 15}$$

$$z_{+2\sigma} = 0.0150(\Delta(\delta D)/8)^4 + 0.738(\Delta(\delta D)/8)^3 + 9.031(\Delta(\delta D)/8)^2 - 47.186(\Delta(\delta D)/8) \quad \text{Eq. 16}$$

$$z_{-2\sigma} = -0.0126(\Delta(\delta D)/8)^4 - 0.580(\Delta(\delta D)/8)^3 - 5.262(\Delta(\delta D)/8)^2 + 89.212(\Delta(\delta D)/8) \quad \text{Eq. 17}$$

which simplifies to

$$z_{mean} = -3.15\times10^{-6}\Delta(\delta D)^4 - 2.19\times10^{-3}\Delta(\delta D)^3 - 0.597\Delta(\delta D)^2 - 89.40\Delta(\delta D) \quad \text{Eq. 18}$$

$$z_{+1\sigma} = 3.66\times10^{-6}\Delta(\delta D)^4 + 1.44\times10^{-3}\Delta(\delta D)^3 + 0.141\Delta(\delta D)^2 - 5.90\Delta(\delta D) \quad \text{Eq. 19}$$

$$z_{-1\sigma} = -3.08\times10^{-6}\Delta(\delta D)^4 - 1.13\times10^{-3}\Delta(\delta D)^3 - 8.22\times10^{-2}\Delta(\delta D)^2 + 11.15\Delta(\delta D) \quad \text{Eq. 20}$$

$$z_{+2\sigma} = 5.57\times10^{-6}\Delta(\delta D)^4 + 2.21\times10^{-3}\Delta(\delta D)^3 + 0.223\Delta(\delta D)^2 - 7.19\Delta(\delta D) \quad \text{Eq. 21}$$

$$z_{-2\sigma} = -5.62\times10^{-7}\Delta(\delta D)^4 + 2.09\times10^{-4}\Delta(\delta D)^3 + 0.181\times10^{-2}\Delta(\delta D)^2 + 35.01\Delta(\delta D) \quad \text{Eq. 22.}$$

This lapse rate can be tested by plotting a set of $\delta^{18}O$ data using the original $\delta^{18}O$ lapse rate from Rowley (2007), then linearly transforming the same $\delta^{18}O$ data to δD using the GMWL. The plots should be identical, but with differing x axes (Figures S1 and S2).

Volcanic glass stable isotopic results were corrected for global changes in ocean chemistry (Zachos et al., 2001) (Figure S3). Following results presented in Zachos et al. (2001), two third order polynomials were used to account for the late Oligocene – Pleistocene relative shift of ~3‰ ^{18}O (~24‰ D) (Figure S3). Specifically, the correction requires adding the difference between the polynomial fit and modern δD‰. Without this correction, all regions yield post-shift δD values that are much more negative the modern range.

References

Craig, H. (1961). Isotopic variations in meteoric waters. Science, 133(3465), 1702-1703.

Rowley, D. B. (2007). Stable isotope-based paleoaltimetry: Theory and validation. Reviews in Mineralogy and Geochemistry, 66(1), 23-52.

Zachos, J., Pagani, M., Sloan, L., Thomas, E., & Billups, K. (2001). Trends, rhythms, and aberrations in global climate 65 Ma to present. science, 292(5517), 686-693.

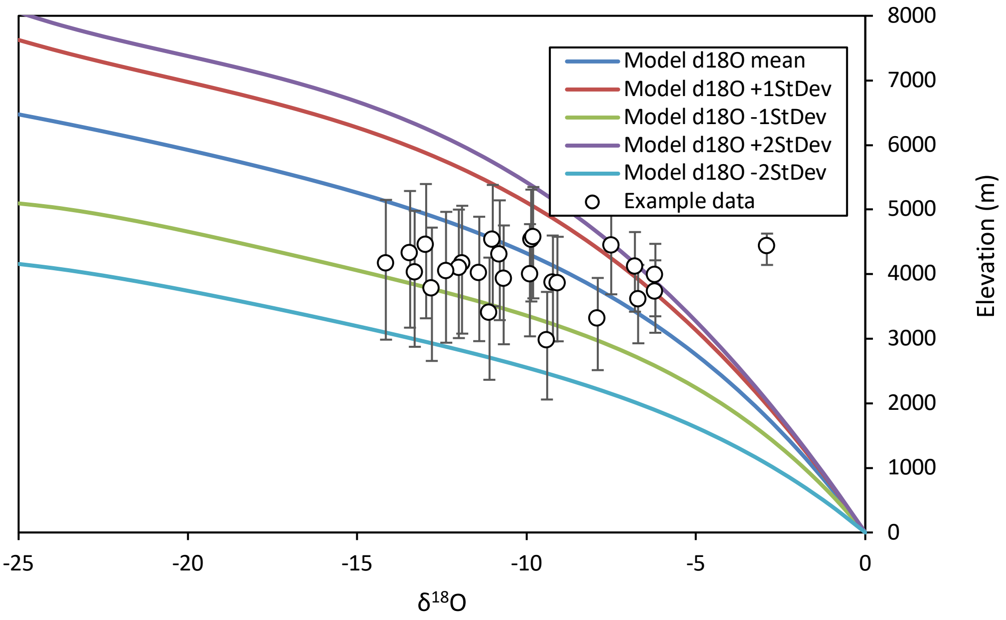

Figure S1. Example $\delta^{18}O$ data vs mean catchment hypsometry.

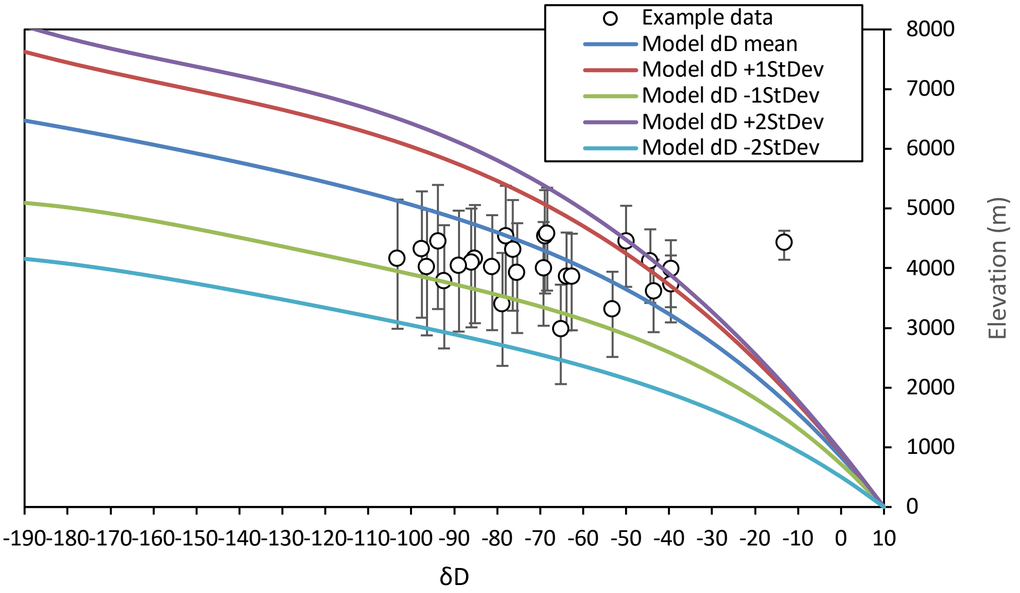

Figure S2. Example $\delta^{18}O$ data converted to δD with the global meteoric water line (Craig, 1961).

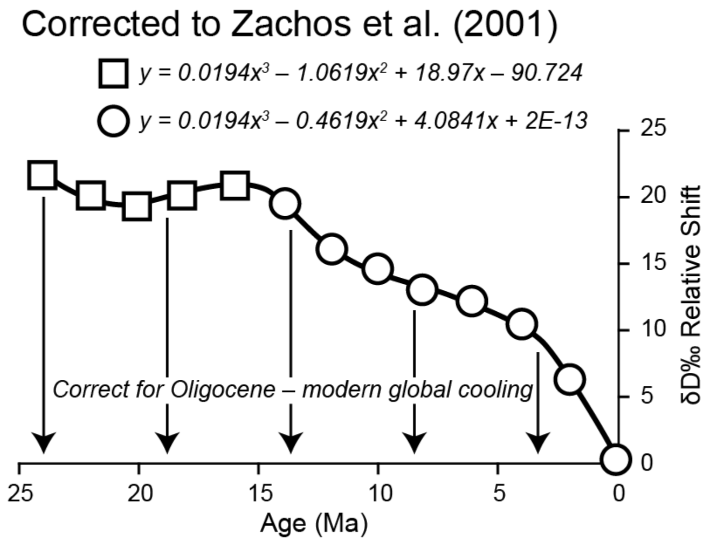

Figure S3. Volcanic glass stable isotopic results were corrected for global changes in ocean chemistry (Zachos et al., 2001). Following results presented in Zachos et al. (2001), two third order polynomials were used to account for the late Oligocene – Pleistocene relative shift of ~3‰ ^{18}O (~24‰ D).

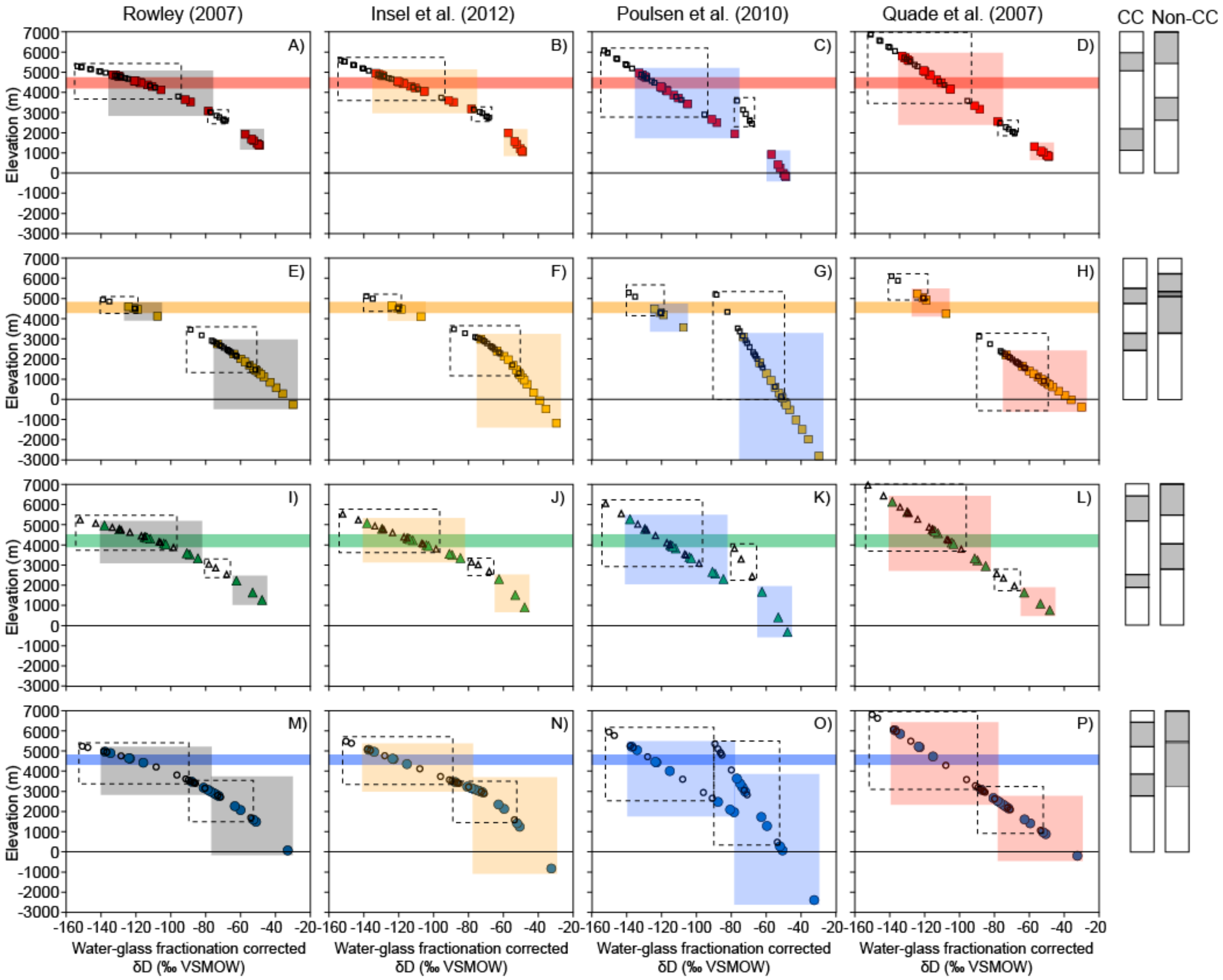

Figure S4. Elevation calculations for each physiographic region based on different lapse rates. Glass δD data are separated into pre-shift (>90‰ δD_{pw}) and post-shift (<90‰ δD_{pw}) water-glass fractionation corrected (Friedman et al., 1993). Colored boxes with colored symbols correspond to the range of elevations predicted by each lapse rate (colors are the same as Figs. 4 and 5 in the main article text). These estimates incorporate a climate correction (CC) that takes into account global cooling since the late Oligocene (Zachos et al., 2001). Dashed boxes use the same lapse rates and same data, but do not include an Oligocene-modern climate correction. Horizontal colored boxes are the range in modern elevation (mean ± 1σ) for each sampling area.

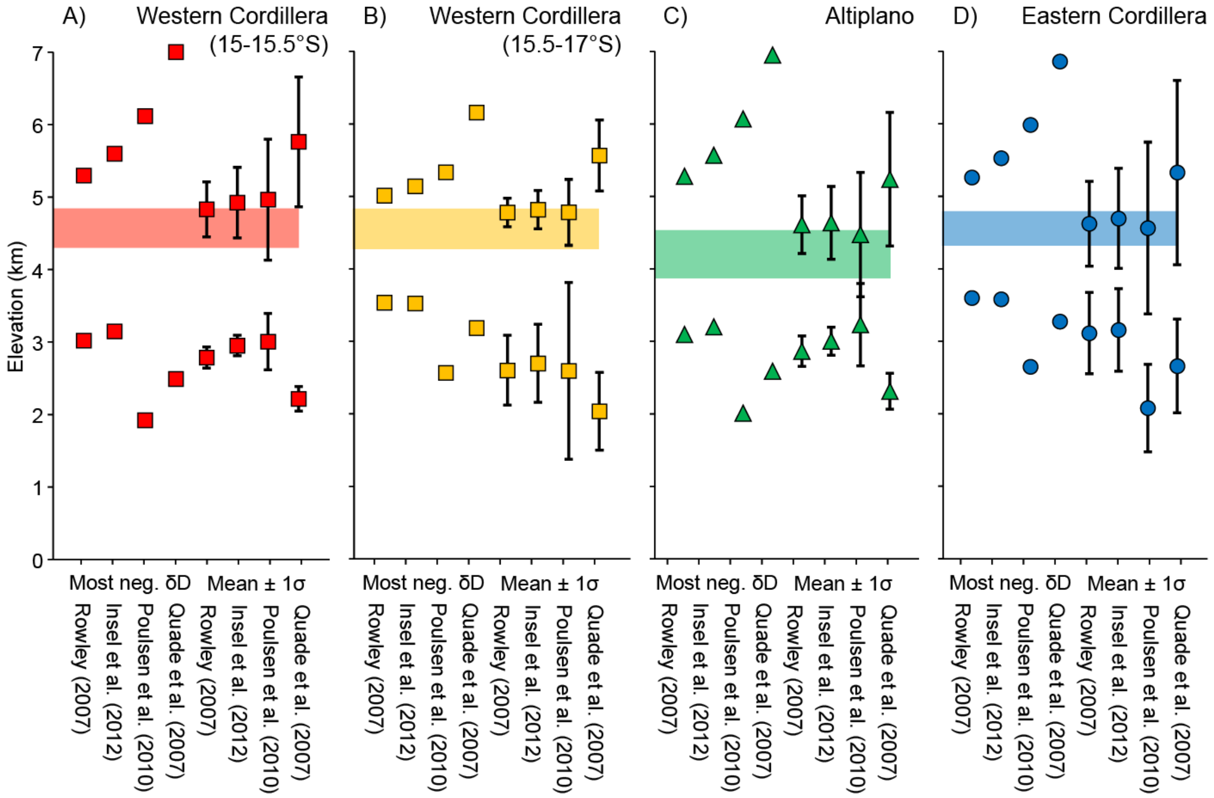

Figure S5. Surface uplift estimates for each physiographic region based on different lapse rates. Estimates *do not* take into account Oligocene – Pleistocene global cooling climate correction. Estimates were calculated using four different lapse rates: thermodynamically-derived non-linear (Rowley, 2007); isotope-tracking general circulation model (GCM) with 25, 50, 75, and 100% of Andean elevations (Insel et al., 2012); GCM with 50 and 100% Andean elevation (Poulsen et al., 2010); and linear empirical (Quade et al., 2007). Surface uplift estimates are calculated based on the most negative and mean pre-shift (> –90‰ δD_{pw}) and post-shift (< –90‰ δD_{pw}) water-glass fractionation corrected δD_{pw} values (Friedman et al., 1993).

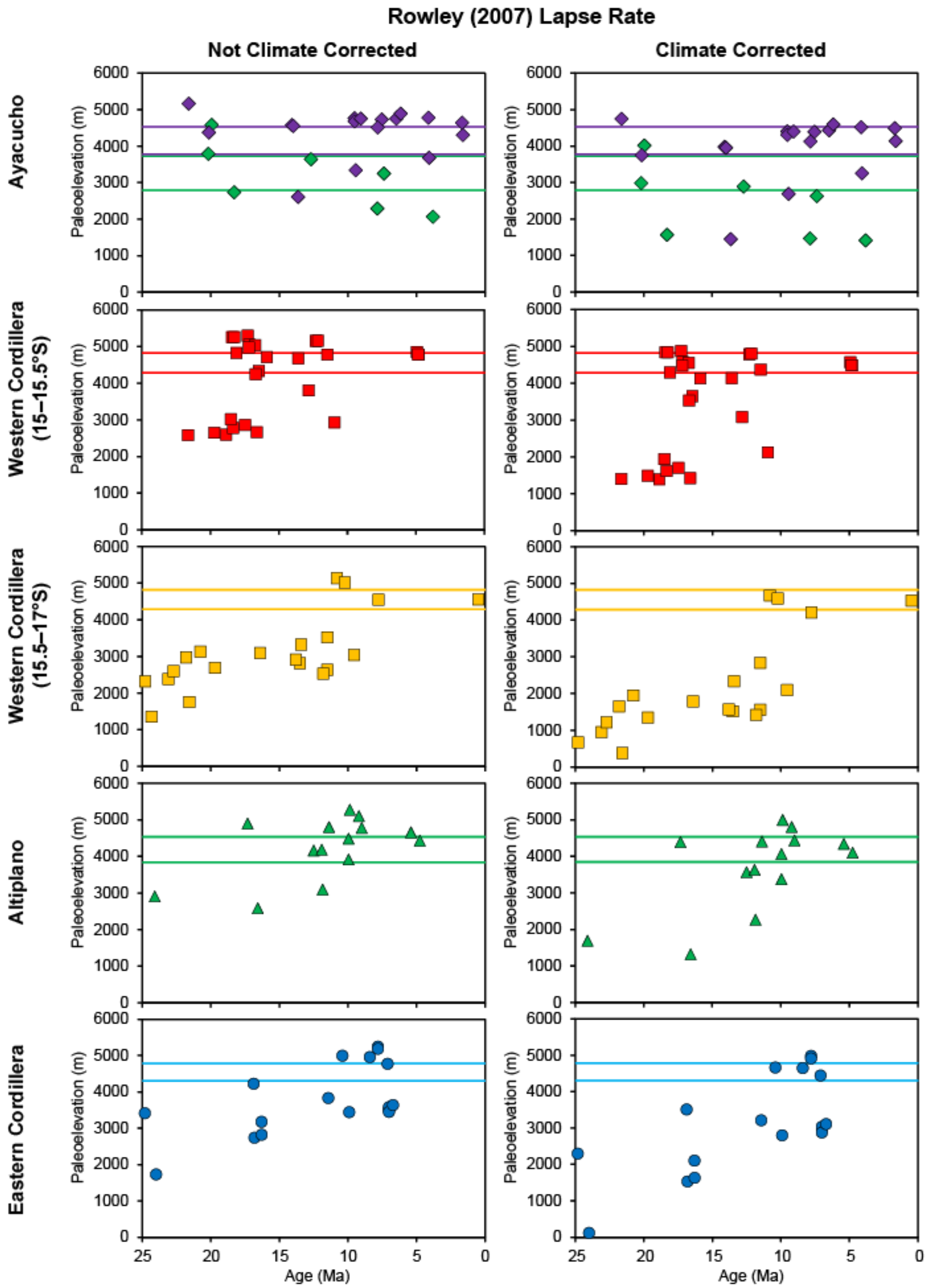

Figure S6. Elevation calculations for each physiographic region based on Rowley (2007) lapse rate. Horizontal bars are ±1σ modern elevation of the sampling areas.

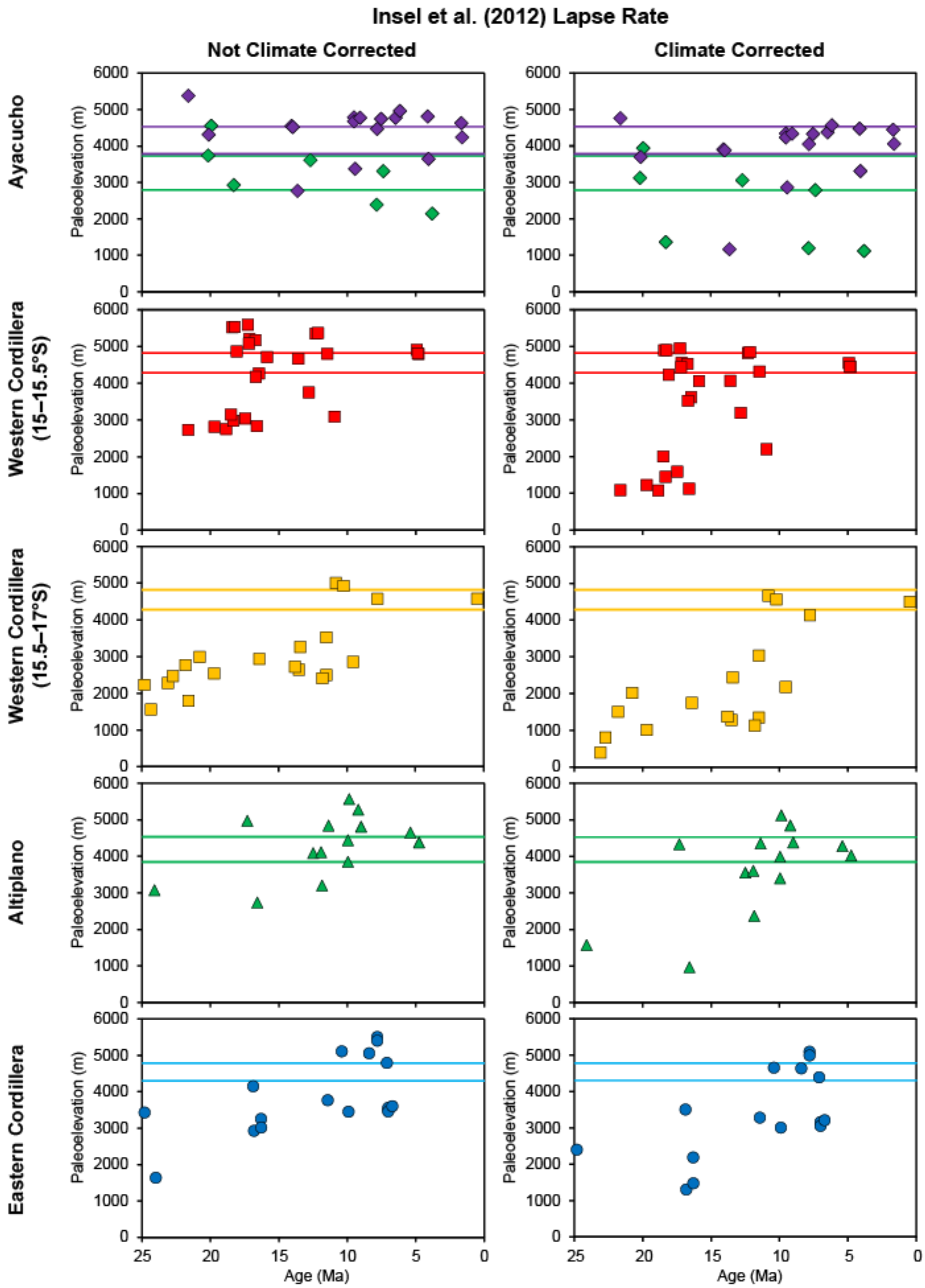

Figure S7. Elevation calculations for each physiographic region based on Insel et al. (2012) lapse rate. Horizontal bars are ±1σ modern elevation of the sampling areas.

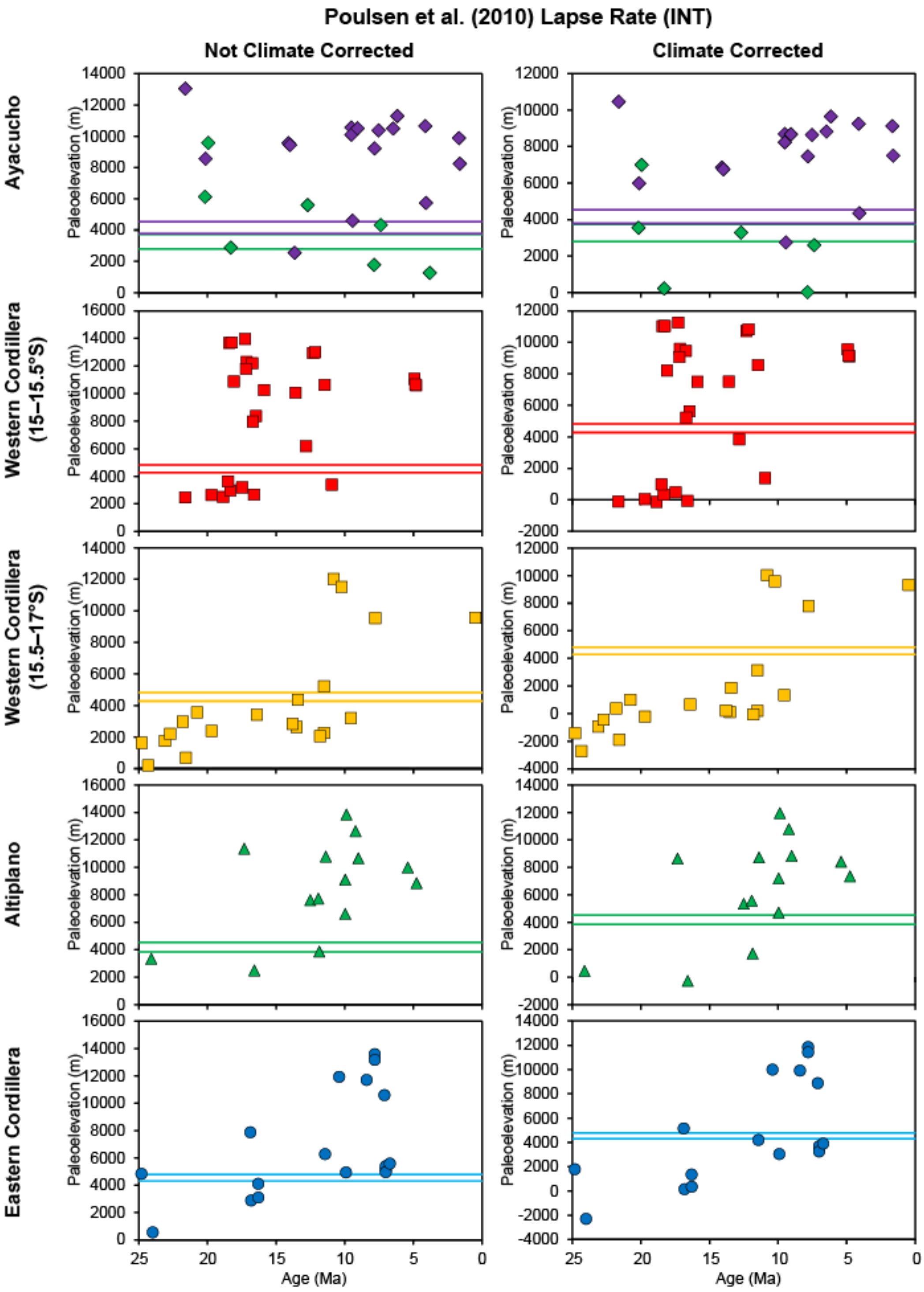

Figure S8. Elevation calculations for each physiographic region based on Poulsen et al. (2010) "INT" lapse rate. Horizontal bars are ±1σ modern elevation of the sampling areas.

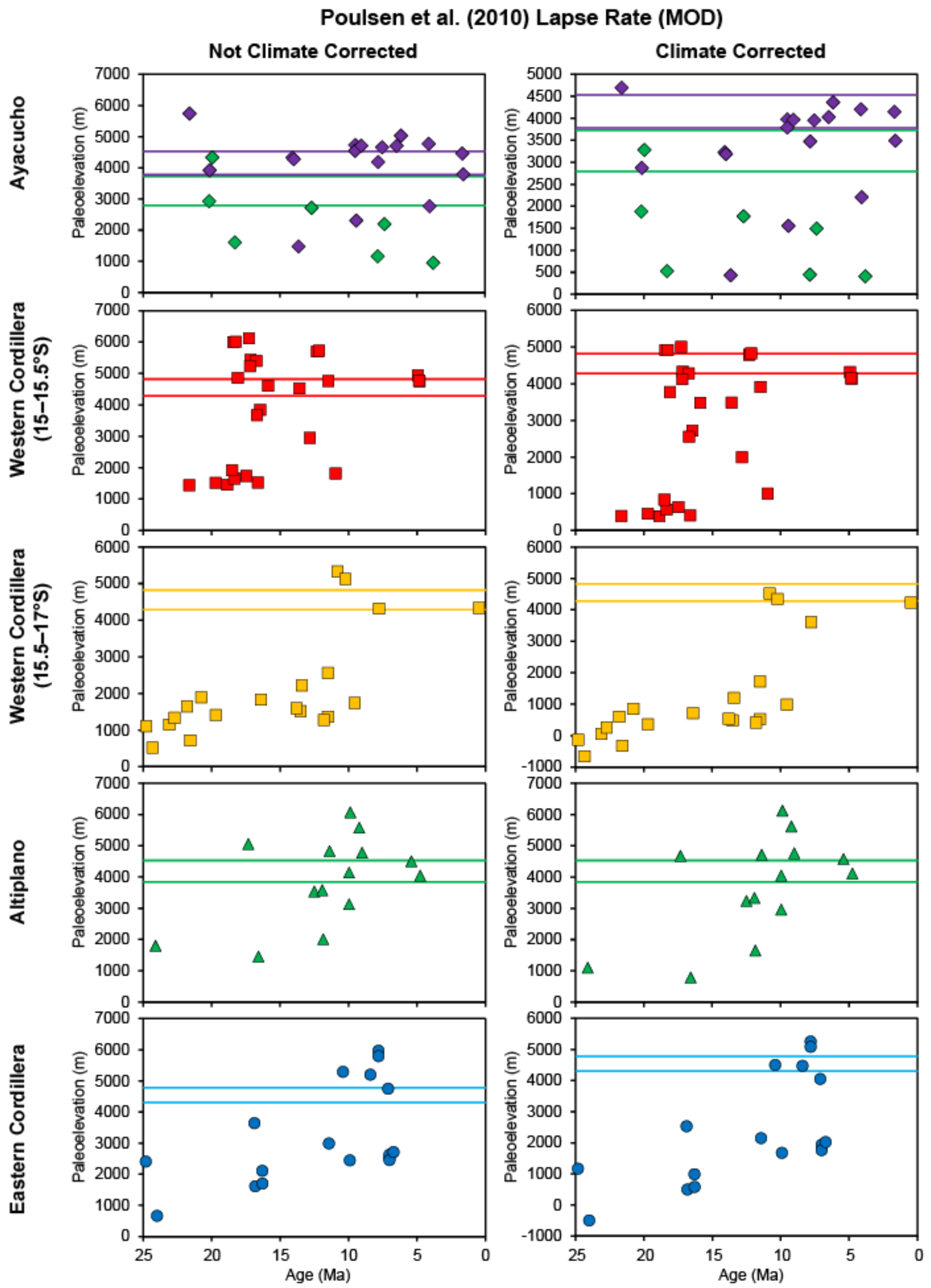

Figure S9. Elevation calculations for each physiographic region based on Poulsen et al. (2010) "MOD" lapse rate. Horizontal bars are ±1σ modern elevation of the sampling areas.

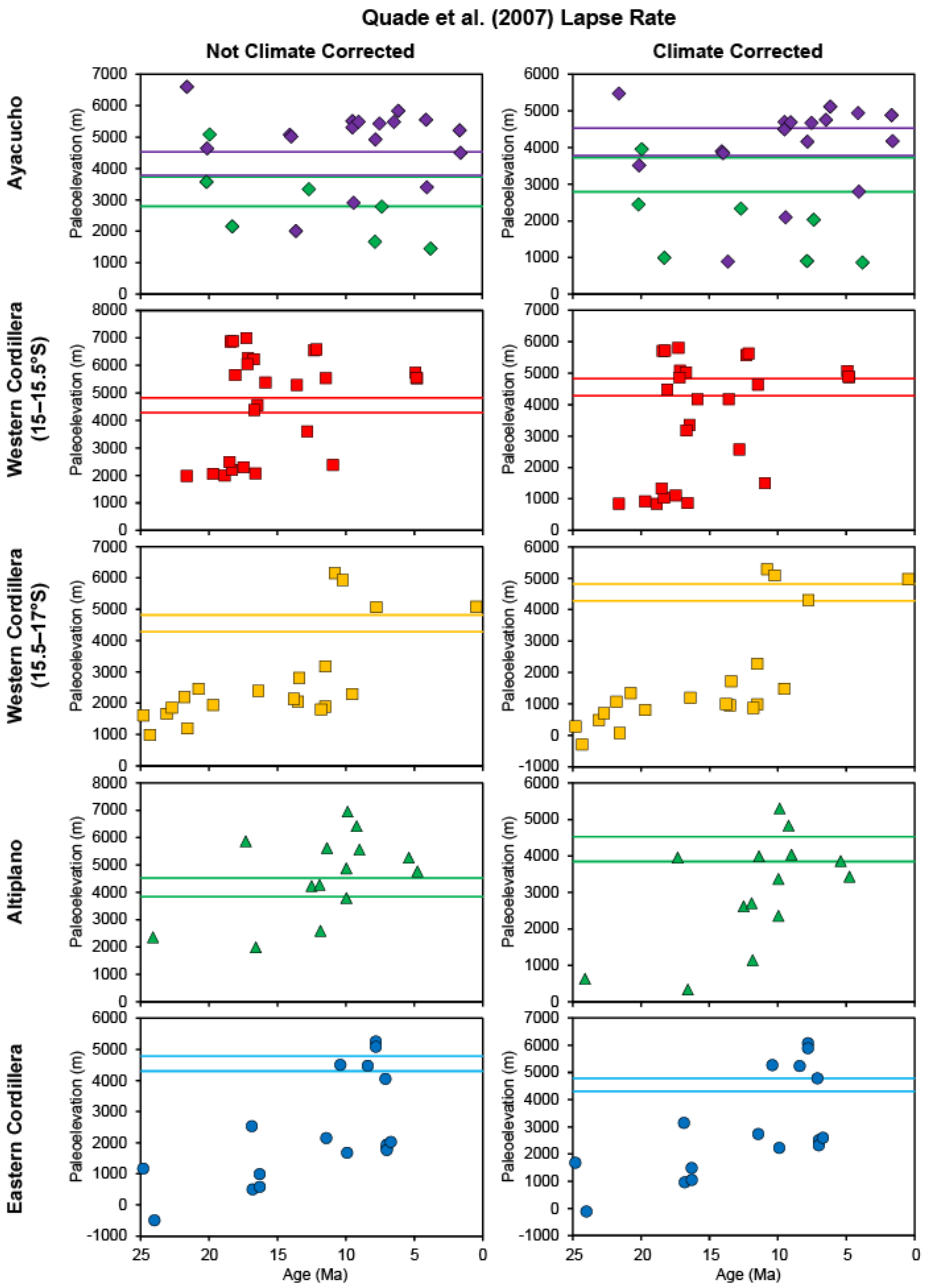

Figure S10. Elevation calculations for each physiographic region based on Quade et al. (2007) lapse rate. Horizontal bars are ±1σ modern elevation of the sampling areas.

Table S1. Surface Uplift Estimates

	Rowley (2007)	Rowley (2007) (Corr.)	Insel et al. (2012)	Insel et al. (2012) (Corr.)	Poulsen et al. (2010)	Poulsen et al. (2010) (Corr.)	Quade et al. (2007)	Quade et al. (2007) (Corr.)
**Western Cordillera (15-15.5°S)**								
Pre-shift maximum	3.0	1.9	3.1	2.0	1.9	0.8	2.5	1.3
Post-shift maximum	5.3	4.9	5.6	4.9	6.1	5.0	7.0	5.8
Difference	***2.3***	***2.9***	***2.4***	***2.9***	***2.5***	***4.0***	***4.5***	***4.5***
Offset from modern	0.7	0.3	1.0	0.4	1.6	0.5	2.4	1.3
Pre-shift mean	2.8	1.7	2.9	1.5	3.0	0.4	2.2	1.1
Pre-shift 1σ	0.1	0.3	0.1	0.4	0.4	0.5	0.2	0.2
Post-shift mean	4.8	4.4	4.9	4.4	5.0	4.0	5.8	4.7
Post-shift 1σ	0.4	0.5	0.5	0.5	0.8	0.8	0.9	0.9
Difference	***2.0***	***2.7***	***2.0***	***2.8***	***2.0***	***3.6***	***3.5***	***3.6***
Offset from modern	0.3	0.2	0.4	0.2	0.4	0.6	1.2	0.2
**Western Cordillera (15.5-17°S)**								
Pre-shift maximum	3.5	2.8	3.5	3.0	2.6	1.7	3.2	2.3
Post-shift maximum	5.0	4.7	5.1	4.7	5.3	4.5	6.2	5.3
Difference	***1.5***	***1.8***	***1.6***	***1.6***	***0.1***	***1.4***	***3.0***	***3.0***
Offset from modern	0.5	0.1	0.6	0.1	0.8	0.0	1.6	0.7
Pre-shift mean	2.6	1.4	2.7	1.2	2.6	0.1	2.0	0.9
Pre-shift 1σ	0.5	0.7	0.5	1.0	1.2	1.4	0.5	0.6
Post-shift mean	4.8	4.5	4.8	4.5	4.8	4.2	5.6	4.9
Post-shift 1σ	0.2	0.2	0.3	0.2	0.5	0.3	0.5	0.4
Difference	***2.2***	***3.1***	***2.1***	***3.3***	***2.2***	***4.1***	***3.5***	***4.0***
Offset from modern	0.2	0.1	0.3	0.1	0.2	0.4	1.0	0.4
**Altiplano**								
Pre-shift maximum	3.1	2.3	3.2	2.4	2.0	1.1	2.6	1.7
Post-shift maximum	5.3	5.0	5.6	5.1	6.1	5.3	6.9	6.1
Difference	***2.2***	***2.7***	***2.4***	***2.8***	***2.2***	***3.6***	***4.4***	***4.5***
Offset from modern	1.1	0.8	1.4	0.9	1.9	1.1	2.8	1.9
Pre-shift mean	2.9	1.8	3.0	1.6	3.2	0.6	2.3	1.2
Pre-shift 1σ	0.2	0.4	0.2	0.6	0.6	0.8	0.2	0.4
Post-shift mean	4.6	4.2	4.6	4.2	4.5	3.7	5.2	4.4
Post-shift 1σ	0.4	0.5	0.5	0.5	0.9	0.9	0.9	0.9
Difference	***1.7***	***2.4***	***1.6***	***2.5***	***1.2***	***3.0***	***2.9***	***3.2***
Offset from modern	0.4	0.0	0.4	0.0	0.3	0.5	1.0	0.2
**Eastern Cordillera**								
Pre-shift maximum	3.6	3.0	3.6	3.2	2.6	1.9	3.3	2.5
Post-shift maximum	5.2	5.0	5.5	5.1	6.0	5.3	6.8	6.1
Difference	***1.7***	***1.9***	***1.9***	***1.9***	***3.3***	***3.3***	***3.6***	***3.6***
Offset from modern	0.7	0.4	1.0	0.5	1.4	0.7	2.3	1.5
Pre-shift mean	3.1	2.1	3.1	2.1	2.1	1.1	2.6	1.6
Pre-shift 1σ	0.6	0.9	0.6	1.2	0.6	0.8	0.6	0.8
Post-shift mean	4.6	4.2	4.7	4.2	4.5	3.8	5.3	4.5
Post-shift 1σ	0.6	0.7	0.7	0.7	1.2	1.2	1.3	1.3
Difference	***1.5***	***2.0***	***1.5***	***2.1***	***2.5***	***2.7***	***2.7***	***2.9***
Offset from modern	0.1	0.4	0.1	0.3	0.0	0.8	0.8	0.1

All estimates are in km. Corr. = Climate corrected for Oligocene - Pleistocene global cooling
Difference represents magnitude of surface uplift.

Table S1. Surface uplift magnitudes based on pre- and post-shift water-glass fractionation-corrected δD_{pw}. Note that the relative surface uplift magnitudes are similar regardless of lapse rate and if the climate correction is applied.

Figure references

Craig, H. (1961). Isotopic variations in meteoric waters. Science, 133(3465), 1702-1703.

Friedman, I., Gleason, J., & Warden, A. (1993). Ancient climate from deuterium content of water in volcanic glass. Climate change in continental isotopic records, 309-319.

Insel, N., Poulsen, C. J., Ehlers, T. A., & Sturm, C. (2012). Response of meteoric δ18O to surface uplift—Implications for Cenozoic Andean Plateau growth. Earth and Planetary Science Letters, 317, 262-272.

Poulsen, C. J., Ehlers, T. A., & Insel, N. (2010). Onset of convective rainfall during gradual late Miocene rise of the central Andes. science, 328(5977), 490-493.

Quade, J., Garzione, C., & Eiler, J. (2007). Paleoelevation reconstruction using pedogenic carbonates. Reviews in Mineralogy and Geochemistry, 66(1), 53-87.

Rowley, D. B. (2007). Stable isotope-based paleoaltimetry: Theory and validation. Reviews in Mineralogy and Geochemistry, 66(1), 23-52.

Zachos, J., Pagani, M., Sloan, L., Thomas, E., & Billups, K. (2001). Trends, rhythms, and aberrations in global climate 65 Ma to present. science, 292(5517), 686-693.
